# Maternal prenatal depressive symptoms and toddler behavior: an umbilical cord blood epigenome-wide association study

**DOI:** 10.1038/s41398-022-01954-6

**Published:** 2022-05-05

**Authors:** Theodora Kunovac Kallak, Emma Fransson, Emma Bränn, Hanna Berglund, Susanne Lager, Erika Comasco, Robert Lyle, Alkistis Skalkidou

**Affiliations:** 1 Department of Women’s and Children’s Health, Uppsala University, Uppsala, Sweden; 2 Department of Women’s and Children’s Health, Science for Life Laboratory, Uppsala University, Uppsala, Sweden; 3 Department of Medical Genetics and Norwegian Sequencing Centre (NSC), Oslo University Hospital, Centre for Fertility and Health, Norwegian Institute of Public Health, Oslo, Norway

**Keywords:** Human behaviour, Clinical genetics

## Abstract

Children of mothers with prenatal depressive symptoms (PND) have a higher risk of behavioral problems; fetal programming through DNA methylation is a possible underlying mechanism. This study investigated DNA methylation in cord blood to identify possible “at birth” signatures that may indicate susceptibility to behavioral problems at 18 months of age. Cord blood was collected from 256 children of mothers who had self-reported on symptoms of depression during pregnancy and the behavior of their child at 18 months of age. Whole genome DNA methylation was assessed using Illumina MethylationEPIC assay. The mother and child pairs were categorized into four groups, based on both self-reported depressive symptoms, PND or Healthy control (HC), and scores from the Child Behavior checklist (high or low for internalizing, externalizing, and total scores). Adjustments were made for batch effects, cell-type, and clinical covariates. Differentially methylated sites were identified using Kruskal–Wallis test, and Benjamini–Hochberg adjusted *p* values < 0.05 were considered significant. The analysis was also stratified by sex of the child. Among boys, we observed higher and correlated DNA methylation of one CpG-site in the promoter region of *TPP1* in the HC group, with high externalizing scores compared to HC with low externalizing scores. Boys in the PND group showed lower DNA methylation in *NUDT15* among those with high, compared to low, internalizing scores; the DNA methylation levels of CpGs in this gene were positively correlated with the CBCL scores. Hence, the differentially methylated CpG sites could be of interest for resilience, regardless of maternal mental health during pregnancy. The findings are in a relatively healthy study cohort, thus limiting the possibility of detecting strong effects associated with behavioral difficulties. This is the first investigation of cord blood DNA methylation signs of fetal programming of PND on child behavior at 18 months of age and thus calls for independent replications.

## Introduction

Peripartum depression refers to a depressive episode starting during pregnancy or postpartum and has a prevalence of 10–20% [1]. Prenatal and neonatal complications, including maternal psychological ill-health, are known risk factors for the development of child psychopathology [2, 3]. Children of mothers affected by peripartum depression, defined by the Diagnostic and Statistical Manual of Mental Disorders—DSM-5 [4] are at increased risk of developing emotional problems [5], such as internalizing or externalizing behavioral problems (reviewed by Stein et al. [6]). Prenatal depression is likely to have different consequences for infant development compared to depressive symptoms with onset in the postpartum period, possibly acting through mechanistic biological pathways of relevance to fetal development [6]. In our recent study on children at 18 months of age, maternal prenatal depressive symptoms (PND) were more predictive of child behavior problems than postpartum depression [7]. Several studies have indicated persistent associations between maternal mental well-being and child outcomes from infancy to teenage years and young adulthood [6, 8]. Notably, elevated levels of externalizing and internalizing behavioral problems in childhood are related to a higher risk of ill-health and sick leave in adulthood [9]. Apart from genetic predisposition, maternal prenatal depression could impact on child development through diverse pathways (such as nutrient, hormonal, and immune pathways), many of which could also influence epigenetic mechanisms [10]. Moreover, altered brain development, i.e., smaller hippocampal volume [11] and greater functional connectivity of the amygdala with the left temporal cortex amygdala microstructure [12], has been suggested in infants exposed to maternal PND and prenatal anxiety. However, the mechanisms of the intergenerational transmission of mental health problems are not fully elucidated [13]. It is also important to note that most children develop normally, and that children exposed to PND can be resilient.

The research field addressing epigenetic programming includes studies on experimental animal models [14] as well as an increasing number of investigations studying the influence of prenatal exposure to maternal depressed mood among infants [15–17]. DNA methylation [18] is a modification that is chemically stable, heritable through DNA replication, can affect gene expression, and is a possible mechanism for long-term consequences of environmental exposure on phenotype [19]. Despite the temporal and cell-specificity of patterns of DNA methylation in the brain and the significant role of epigenetics in neuronal development [20], more easily accessible tissues are currently being investigated to assess whether they could provide evidence for the methylation status in the brain. Peripheral blood has been demonstrated as a valid surrogate tissue for the brain in epigenome-wide association studies (EWAS) [21].

Furthermore, the sex of the child may also moderate the epigenetic pathways in relation to child development, since there are differences between sexes in DNA methylation patterns [22] and also how child behavior is impacted by PND [7, 23]. Importantly, cord blood differs in cell composition in comparison to whole blood from adults [24, 25], and previous studies on the association between PND and DNA methylation in cord blood have not taken this into account [16, 17].

EWAS in cord blood could increase our understanding of the biological mechanisms underlying the impact of exposure to depressive symptoms during pregnancy on child development. A recent review investigating the possible role of DNA methylation as a mediator in the association between PND and child behavior showed only a few studies investigating exposure, mediator, and child outcomes in the same study cohort with a longitudinal study design [26]. Previous studies have found associations between PND and cord blood DNA methylation in candidate genes [16, 17, 27], whilst findings by use of EWAS have shown varied results [15, 28]. Notably, findings in the Avon Longitudinal Study of Parents and Children could not be replicated in the Generation R [28]. Hypo-methylation in *ZNF575*, a gene whose functions are largely unknown, has been found in newborns exposed to antidepressants during fetal life and confirmed in two study cohorts [15]. Interestingly, the hypo-methylation persisted into early childhood [15]. We have recently shown that PND alone does not affect cord blood DNA methylation when adjusting for cell composition, covariates, and using stratified analyses by sex of the child. We did, however, identify several genes associated with brain development and function, differentially methylated in those who were exposed to selective serotonin reuptake inhibitors (SSRIs) in comparison with untreated PND and healthy controls (HC) [29]. Nonetheless, the above-mentioned studies did not investigate the behavior of the child later in life. Cross-sectional studies on DNA methylation of candidate genes, in whole blood and saliva, have shown an association with internalizing behavior [30], with depressive symptoms in mother and child [31], and also with later development of autism [32].

However, to the best of our knowledge, no study has jointly considered exposure to PND, DNA methylation in cord blood, and longitudinal follow-up of child behavior, when also taking into account possible sex differences and blood cell composition. Our longitudinal study design also enables the possibility to exclude participants with depressive symptoms in the postpartum period, thereby excluding the possible impact of postpartum depressive symptoms on child behavior in an attempt to look at DNA methylation as a mediator of the association between antenatal depression and child psycho-emotional development.

The aim of the present study, therefore, was to investigate if DNA methylation differences in cord blood could serve as an early marker for future development of behavioral problems at the age of 18 months, taking into account the exposure to maternal PND and SSRI use, as well as possible confounders and sex of the child.

## Methods

### Study population

Mothers and children participating in the BASIC Study (Biology, Affect, Stress, Imaging, and Cognition in pregnancy and puerperium) and in the follow-up study, U-BIRTH, were included in this study. The BASIC study is a longitudinal project that recruited pregnant women between 2009 and 2018 with the aim of enhancing the knowledge of prenatal and postpartum depression [33]. U-BIRTH focused on the psychosocial development of the children born to mothers in the BASIC cohort [7]. Both studies were conducted at the Department of Women’s and Children’s Health, Uppsala University and the Women’s clinic at the Uppsala University Hospital. Ethical approval was granted from the Uppsala Ethical Review Board reference numbers, BASIC 2009/171 with amendments and U-BIRTH 2012/010 with amendments.

The BASIC study invited all pregnant women in Uppsala County, who were over 18 years of age, speaking Swedish, and scheduled for a routine ultrasound at Uppsala University Hospital. Women diagnosed with a pathological pregnancy (malformations leading to termination of pregnancy or miscarriage), with blood-borne disease, or with protected personal data were excluded. Approximately 22% of all women giving birth at the time during the inclusion period in Uppsala County were estimated to participate in the study cohort [33]. All participating women gave signed consent for all parts of the study. Participants contributed via web-based questionnaires during gestational weeks 17 and 32 and at 6 weeks, 6 and 12 months after giving birth. Cord blood was collected during childbirth. Participants who accepted to participate in the follow-up study (U-BIRTH) received a questionnaire when their children had reached 18 months of age. Approximately 50% of the BASIC participants were enrolled in the follow-up study.

Participating mothers reported their age, education, smoking habits, employment, and country of birth (a proxy for ethnicity), including medical history for depressive symptoms, by use of web-based questionnaires at gestational week 17. Medical records were used to extract medical information about the pregnancy and childbirth.

The Swedish validated version of the Edinburgh Postnatal Depression Scale (EPDS) [34, 35] was used in the web-based questionnaires at pregnancy weeks 17 and 32. Depending on the time point of inclusion to the BASIC study [33], anxiety symptoms were measured with the State-Trait Anxiety Inventory for Adults (STAI-AD using the state scale) or the Beck Anxiety Inventory (BAI). If the participant did not fill out any of these or if she had internal missing data, anxiety symptoms were based on the EPDS anxiety subscale [36]. When the child was 18 months of age, participating mothers were asked to fill out the Child Behavior Checklist (CBCL) 1 ½–5. The CBCL can be summarized into either a total score ranging from 0 to 200, with higher scores indicating more problematic behaviors or, based on subsets of the questions, into two subscales for externalizing (ranging from 0 to 48 points) and internalizing (ranging from 0 to 72 points) child problems [37]. In this study, the participants were categorized according to their CBCL score to high CBCL (median and above) and low CBCL (scores in the 5th–40th percentile); participants in the 0–5th and 40th–50th percentiles were excluded from the analysis.

Mothers and children in this sub-study were included if they had been followed throughout the pregnancy and the postpartum period, if a cord blood sample was collected at birth, and if the mother-child pair also participated in the follow-up study. Exclusion criteria were depressive symptoms present in the postpartum period and maternal smoking during pregnancy, as these variables affect the psycho-emotional development and epigenetic modifications, respectively. If a woman had participated multiple times in the BASIC project, only one pregnancy was included. If the mother experienced depressive symptoms in one pregnancy but not the other, the one with depressive symptoms was chosen; otherwise, the first pregnancy was included. Mothers defined as HC had to have answered all questionnaires throughout the study, had EPDS ≤ 11 at both pregnancy weeks 17 and 32. They were excluded if they had anxiety, defined by the use of STAI-AD, BAI, or the EPDS anxiety subscale, any history of depression, or missing information in their medical records. Women with depressive symptoms during pregnancy (PND) were identified by the use of EPDS > 12. Women with pruritus, placenta previa, anemia, hepatosis, cystitis, preeclampsia, or HELLP syndrome were excluded. This resulted in 256 mother-child pairs being included in the final analysis.

**Fig. 1:**
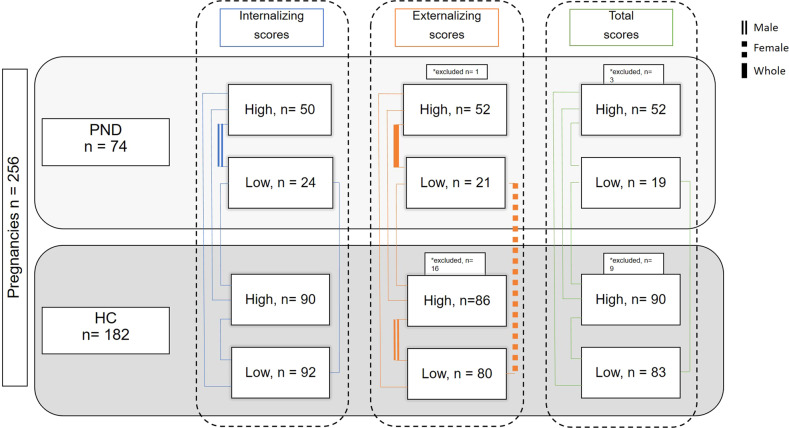
Groups for comparison of differentially methylated (DM) CpGs in association with Child Behavior Checklist (CBCL) scores and maternal mood assessed by use of the Edinburgh Postnatal Depression Scale. Results with DM CpGs with significant adjusted *p* value are marked by bold lines for the whole study population, by dotted lines when considering only female children, and by double lines when considering only male children. PND perinatal depression, HC healthy control, High CBCL scores median and above, Low CBCL scores 5–40 percentile. *Excluded due to below percentile and between 41 percentile to median.

In mother and child pairs, irrespective of maternal mood, all children with high (median and above) were compared to all children with low (5th percentile–40th percentile) for CBCL internalizing problems, externalizing problems, and total scores. In addition, mother and child pairs were further categorized based on maternal mood, HC or PND, and on self-reported CBCL scores high (median and above) or low (5th percentile–40th percentile) for internalizing problems, externalizing problems, and total scores, resulting in four study groups for each of the three CBCL summarized score analyses, as presented in Fig. 1.

### Cord blood collection and DNA extraction

For this study, whole blood from the cord vein was collected at birth. The samples were stored at −70 °C until further processing. DNA was extracted from the samples with the QIAamp DNA blood/mini kit (Qiagen), following the manufacturer’s instructions. Concentration and purity of the extracted DNA were measured with a NanoDrop Spectrophotometer (Thermo Scientific).

Throughout the experimental procedure (from DNA extraction to DNA methylation analysis), samples from the PND and the HC groups were mixed in order to prevent detecting batch effects.

### DNA methylation data

The Zymo Research DNA Methylation-Lightning Kit (Zymo Research) was used for bisulfite conversion of DNA. Illumina MethylationEPIC kit (Illumina) was thereafter used for genome-wide methylation analysis at ~850,000 CpG sites. All reactions were performed according to the manufacturer’s instructions at Life & Brain Research Centre, University Hospital of Bonn, Germany. Laboratory personnel were blinded to the group identity of the samples during the analysis. National Bioinformatics Infrastructure Sweden was employed to perform the data processing in R 3.5.2 using R package minfi 1.28.4 [38]. The workflow followed the recommendations by the tool guide. Detection *p* value (DPV) with a cut-off at 0.01 was used to remove CpGs with poor signal (4.5% of the CpGs had DPV > 0.01 in 10% or more of the samples). No samples, but only individual CpGs, were removed based on the DPV. As recommended by the Illumina MethylationEPIC kit (Illumina), ~3.2% of the CpGs were rejected due to presence of known SNPs in the CpG site or single base extension. SNP annotation data were obtained through minfi:getSnpInfo, which retrieves SNP data from IlluminaHumanMethylationEPICanno.ilm10b2.hg19 version 0.6.0.

Quantile normalization, suitable for datasets where global differences are not expected between samples [39], was performed by use of functions in the R package minfi. Information about sex was provided to a function (rgf_quantile ← preprocessQuantile(rgf,sex = sx), beta_quantile ← getBeta(rgf_quantile), m_quantile ← get_mv(beta_quantile), presented by Touleimat and colleagues, which implements a stratified quantile normalization that is applied to the methylated and unmethylated signal intensities separately, and takes into account the different probe types [40]. This method corrects for non-biological variation between CpGs. Thereafter, the data were transformed into beta, representing the proportion of cells methylated at the each CpG site (from 0 to 1 methylation) and *M* values for statistical analysis. *B* values were chosen for visualization, as recommended in the literature [41].

According to the tool guide, CpGs from the X and Y chromosomes were discarded, and only autosomal CpGs were used for downstream analyses. Cell composition in each sample was estimated using R package FlowSorted.CordBlood.450k [25] as the reference data and thereafter included in the models.

### Statistical analyses

Principle component analysis (PCA) was used in exploratory data analyses to investigate the general structure and patterns in the dataset by using Beta values of the 100,000 CpGs with the highest variance. Up to 17% of the total variation in the dataset was explained by the first three principal components (PCs). Spearman correlation and Kruskal–Wallis test, both corrected using Benjamini–Hochberg method, were used to investigate the associations of the top 6 PCs with categorical and continuous variables, respectively, of interest.

Even though PCA were done on 100,000 with the highest variance, group comparisons to identify differential methylation (DM) CpGs were performed by use of R package Limma on 813,685 CpGs. The data were analyzed all together or stratified by sex of the child and finally also excluding participants using SSRI due to our previous findings [29]. *M* values were used for the DM analysis. For each group comparison, a linear model was built and run crude (unadjusted (0 covariates)), adjusted for only technical variables (5 covariates), or also in combination with clinical characteristics that might impact on perinatal depression, DNA methylation, and CBCL scores (12 covariates in total). Five technical variables were included in the model: sample plate batch, sentrix position on the chip, granulocyte cell estimate, red blood cell estimate, and CD8 T cell estimate [25, 42]). Also, clinical characteristics such as maternal age at delivery, BMI before pregnancy, maternal place of birth (Scandinavia or other), educational level (University degree yes or no), mode of delivery (vaginal, vacuum extraction, emergency cesarean section, and elective cesarean section), parity, and gestational age, based on previous literature [15, 26, 43–45], were also included in the model, in total 12 covariates. Results are presented for the model based on adjustments for 5 and 12 covariates. Differential DNA methylation between groups in specific CpGs was presented as mean and standard deviation and log2 fold change (log2 (*M* value group 1/*M* value group 2)) between the groups, meaning that a negative log2 fold changes equals lower DNA methylation in group 1. Identified CpGs were annotated by use of the CpG sites of interest which were annotated to genes, genomic regions, and chromosomal and map locations with the UCSC genome browser in accordance with GRCH37/hg19 and further investigated by use of genecards.org and the EWAS Atalas http://bigd.big.ac.cn/ewas.

In addition to group comparisons using linear modeling, R package Limma was used to conduct regression models. The regression models allowed us to analyze the association between DNA methylation in identified significantly DM CpGs and CBCL scores. The CBCL scores were used as continuous variables with or without using continuous EPDS as an interactor. The models were adjusted for 0, 5, or 12 covariates.

Genes of interest previously identified in the literature in studies investigating PND and DNA methylation [15, 17, 27, 31] were also analyzed separately. In this analysis, adjustment of the *p* value for multiple comparisons was based on the number of CpGs in the genes of interest, according to the Illumina manifest. Included genes of interest were: nuclear receptor subfamily 3 group c member 1 (*NR3C1*), collagen type VII alpha 1 chain (*COL7A1*), zinc finger protein 575 *(ZNF575*), maternally expressed gene 3 (*MEG3*), solute carrier family 6 member 4 (*SLC6A4*), hydroxysteroid 11-beta dehydrogenase 2 (*HSD11B2*), FKBP prolyl isomerase 5 (*FKBP5*), brain derived neurotrophic factor (*BDNF*), insulin like growth factor 2 (*IGF2*), PLAG1 like zinc finger 1 (*PLAGL1*), mesoderm specific transcript (*MEST*), and paternally expressed 3 (*PEG3*) [15, 17, 27, 31].

Lastly, to investigate if the methylation profile for the selected genes of interest can predict the child behavior at 18 months of age, three machine learning models were applied: logistic regression with lasso regularization [46] (implemented in the R-package glmnet), random forest (RF) [47] (R-package randomForest), and partial least squares-discriminant analysis (PLS-DA) [48] (R-package ropls).

The classifiers were trained on 80% of the data and tested on the remaining 20% in 50 repeated holdouts. In order to avoid unequal class sizes, an equal number of samples from each class was selected for training and evaluation. For each model, the average accuracy (average over the 50 holdouts) was computed. For each machine learning model, the outcome variables (CBCL internal scores, external scores, total scores) and a subset of data were used for both a model based on only covariates (age, BMI, place of birth, parity, gestational age, mode of delivery, and education) as well as a model based on covariates and methylation sites. This was performed in the whole cohort and then stratified by sex of the child (see Fig. 1). The analyses were repeated using: (1) the four subgroups based on the CBCL scores, (2) CBCL scores only irrespective of maternal mood, (3) only children in the PND low CBCL and high CBCL groups, and (4) only HC low CBCL and high CBCL groups. Finally, to investigate the possible epigenetic markers of resilience or susceptibility respectively, one analysis was performed including only the extreme groups (5) PND low CBCL and HC high CBCL. The methylation data were adjusted for technical variables (sample plate batch, sentrix position on the chip, granulocyte cell estimate, red blood cell estimate, CD8 T cell estimate) before modeling.

## Results

### Demographics

Due to the definition of high and low CBCL scores, meaning high (median and above) or low (5th percentile–40th percentile), different numbers of mother-child pairs were included in the three different CBCL summarized scores (internalizing, externalizing, and total score), described in Fig. 1. Characteristics of the participating mother and child pairs presented in Table 1 are based on groups for internalizing problem scores. The clinical characteristics did not differ when groups were based on externalizing problem scores or total scores. There were no significant differences in the proportion of mothers born in Scandinavia between any of the analyzed study groups. In general, a slight difference was noted in maternal age, educational level, and parity between groups.

**Table 1:** Characteristics of the participating mother and child pairs.

	Healthy controls, high internalizing score *n* = 90	Healthy controls, low internalizing score *n* = 92	Prenatal depressive symptoms, high internalizing score *n* = 50	Prenatal depressive symptoms, low internalizing score *n* = 24
Maternal characteristics				
Age, median years (range)	31.0 (23.0–41.0)^a^	32.5 (20.0–48.0)^b^	30.0 (19.0–39.0)	31.0 (24.0–40.0)
Born in Scandinavia, *n* (%)^d^	83 (92.2)	88 (95.7)	41 (93.1)	22 (95.6)
Educational attainment, university level, *n* (%)^d^	79 (87.8)	83 (90.2)	13 (56.5)	33 (75.0)^a^
Primipara, *n* (%)	55 (61.1)^a^	41 (44.6)	29 (58.0)	8 (33.3)
Body mass index (BMI), median (range)^e^	22.5 (18.3–33.3)	22.7 (17.7–34.6)	22.4 (18.3–36.7)	24.5 (17.6–36.8)
Vaginal delivery, *n* (%)	74 (82.2)	69 (75.0)	19 (79.2)	39 (78.0)
History of depression, *n* (%)^d^	0^b,c^	0^b,c^	17 (77.3)	34 (77.3)
Anxiety during pregnancy	0^b,c^	0^b,c^	29 (58.0)	13 (54.2)
EPDS, scores at gestational week 17, median (range)^d^	3.0 (0.0–10.0)^b,c^	2.0 (0.0–10.0)^b,c^	13.0 (0.00–21.0)	13.0 (1.0–18.0)
EPDS, scores at gestational week 32, median (range)^d^	3.0 (0.0–11.0)^b,c^	2.0 (0.0–10.0)^b,c^	12.5 (2.00–20.0)	13.0 (5.0–16.0)
SSRI treatment *n* (%)	0^b,c^	0^b,c^	4 (8.0)	3 (12.5)
Child characteristics				
Sex				
Boy	47 (52.2)	43 (46.7)	20 (40.0)	10 (41.7)
Girl	43 (47.8)	49 (53.3)	30 (60.0)	14 (58.3)
Gestational length (days)	281 (258–296)	281 (245–296)	280 (263–296)	282.5 (265–296)
Birth weight (kg) mean (SD)	3.58 (0.49)	3.69 (0.50)	3.59 (0.55)	3.71 (0.49)
Child behavior checklist scores, median (range)				
Internalizing scores	41.0 (39.0–52.0)^a,c^	37.0 (36.0–38.0)^b,c^	42.0 (39.0–53.0)	37.5 (36.0–38.0)
Externalizing scores	32.5 (24.0–51.0)^a,c^	28. 0 (23.0–42.0)^b,c^	36.0 (25.0–56.0	29.5 (24.0–38.0)
Total problem scores	83.5 (70.0–110.0)^a,b,c^	73.5 (67.0–92.0)^b,c^	87.0 (73.0–119.0)	76.5 (68.0–87.0)

*EPDS* Edinburgh Postnatal Depression Scale, *SSRI* selective serotonin reuptake inhibitors. ^a^Significantly different than healthy controls, low internalizing scores. ^b^Significantly different than prenatal depressive symptoms, high internalizing scores. ^c^Significantly different than prenatal depressive symptoms, low internalizing scores. ^d^Data missing on seven subjects. ^e^Data missing on six subjects.

### DNA methylation

**Fig. 2:**
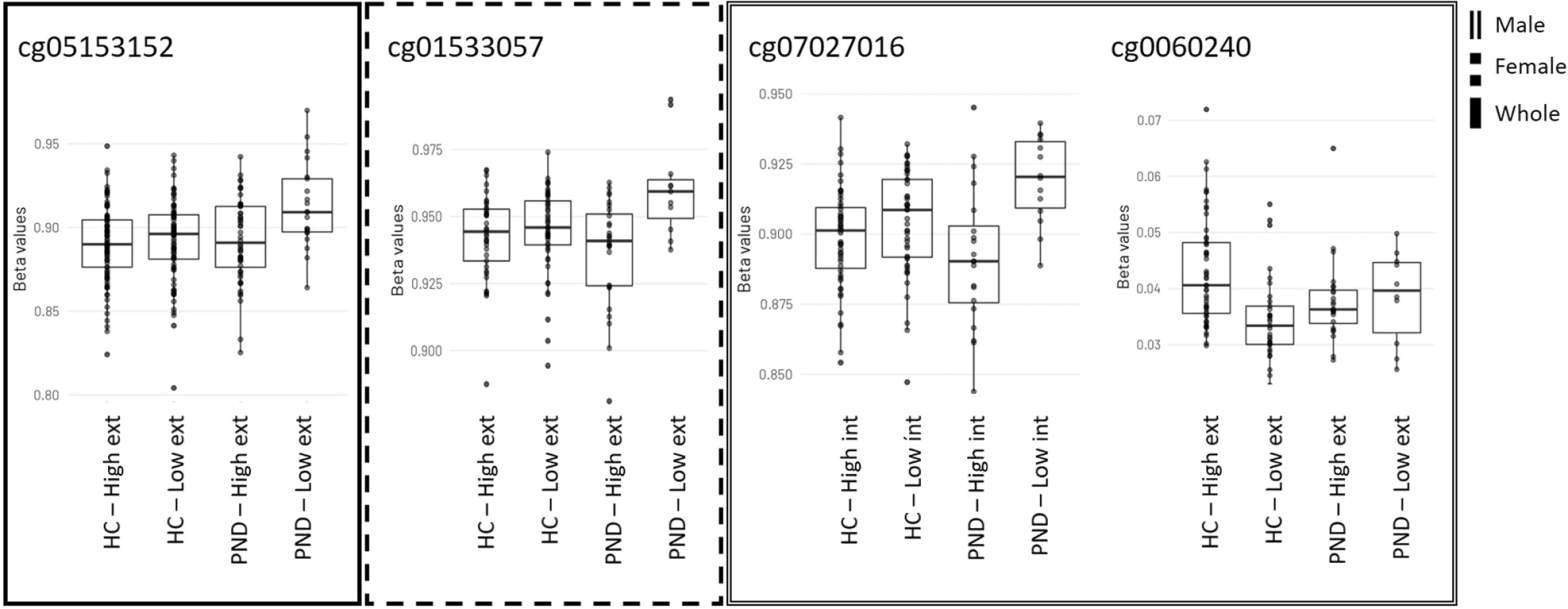
Box plots displaying group-wise distribution (median and percentiles) of beta values of differentially methylated CpGs in the whole study cohort and stratified by sex of the child. PND Perinatal depression, HC healthy control, High CBCL scores median and above, Low CBCL scores 5–40 percentile, int internalizing scores, and ext externalizing scores.

After adjusting for 12 covariates and controlling for multiple testing, the overall number of DM CpG sites between the different study groups is displayed in Table 2, and the number of differentially methylated CpGs is illustrated in Fig. 1. The data are also displayed as quantile-quantile plots in Supplementary Fig. 1. Specific DM sites are shown in Fig. 2 and Supplementary Fig. 2, and in Table 3 with annotated gene, chromosomal location, mean standard deviation for each group and log2 fold change between study groups, and lastly, the regulatory features of the site. Group comparisons adjusted for 0, 5, or 12 covariates are presented in Supplementary Table 1, and DM CpGs identified adjusted for five covariates are presented in Supplementary Table 2.

**Table 2:** Overview of the number of differentially methylated CpG sites in different group comparisons.

		Whole								Female								Male							
		All				SSRI-treatment excluded				All				SSRI-treatment excluded				All				SSRI-treatment excluded			
Group 1	Group 2	*n*1	*n*2	pa	pu	*n*1	*n*2	pa	pu	*n*1	*n*2	pa	pu	*n*1	*n*2	pa	pu	*n*1	*n*2	pa	pu	*n*1	*n*2	pa	pu
High int	Low int	132	115	0	47,111	128	112	0	47,505	70	59	0	34,518	67	58	0	34,590	62	56	0	47,403	61	54	0	46,870
HC-High int	HC-Low int	89	92	0	40,165	89	92	0	40,506	43	49	0	33,980	43	49	0	33,361	46	43	0	39,394	46	43	0	40,542
HC-High int	PND-High int	89	43	0	44,070	89	39	0	41,104	43	27	0	35,021	43	24	0	33,855	46	16	0	44,308	46	15	0	42,135
HC-High int	PND-Low int	89	23	0	71,787	89	20	0	67,190	43	10	0	42,357	43	9	0	39,869	46	13	0	47,373	46	11	0	45,661
HC-Low int	PND-High int	92	43	0	37,819	92	39	0	37534	49	27	0	33217	49	24	0	33340	43	16	0	44808	43	15	0	42288
HC-Low int	PND-Low int	92	23	0	51,457	92	20	0	49,173	49	10	0	38,283	49	9	0	37,605	43	13	0	38,183	43	11	0	36,319
PND-High int	PND-Low int	43	23	0	50,610	39	20	0	52,091	27	10	0	36,241	24	9	0	37,936	16	13	**1**	49,576	15	11	0	45,477
Hight ext	Low ext	134	97	0	37,800	128	95	0	38,268	64	58	0	43,770	60	58	0	41,719	70	39	0	39,338	68	37	0	40,561
HC-High ext	HC-Low ext	85	80	0	44,378	85	80	0	44,866	37	49	0	40,449	37	49	0	40,883	48	31	**1**	46,230	48	31	**1**	46,992
HC-High ext	PND-High ext	85	49	0	69,903	85	43	0	59,957	37	27	0	53,075	37	23	0	44,726	48	22	0	50,427	48	20	0	46,387
HC-High ext	PND-Low ext	85	17	0	37,679	85	15	0	35,903	37	9	0	39,569	37	9	0	38,231	48	8	0	38,968	48	6	0	35,516
HC-Low ext	PND-High ext	80	49	0	50,085	80	43	0	44,444	49	27	0	48,901	49	23	0	42,005	31	22	0	36,059	31	20	0	35,834
HC-Low ext	PND-Low ext	80	17	0	42,682	80	15	0	40,010	49	9	**1**	40,581	49	9	0	37,974	31	8	0	38,662	31	6	0	35,611
PND-High ext	PND-Low ext	49	17	**1**	48,940	43	15	**1**	45,621	27	9	0	60,152	23	9	0	49,011	22	8	0	40,102	20	6	0	37,058
High tot	Low tot	136	100	0	37,124	132	97	0	37,850	67	54	0	37,030	64	54	0	35,779	69	46	0	44,327	68	43	0	41,705
HC-High tot	HC-Low tot	89	83	0	42,788	89	83	0	43,490	41	45	0	33,911	41	45	0	34,068	48	38	0	43,278	48	38	0	43,599
HC-High tot	PND-High tot	89	47	0	62,034	89	43	0	55,409	41	26	0	42,880	41	23	0	38,093	48	21	0	46,215	48	20	0	45,373
HC-High tot	PND-Low tot	89	17	0	42,263	89	14	0	35,599	41	9	0	35,517	41	9	0	35,286	48	8	0	41,582	48	5	0	29,787
HC-Low tot	PND-High tot	83	47	0	42,190	83	43	0	39,766	45	26	0	41,780	45	23	0	37,415	38	21	0	40,619	38	20	0	40,074
HC-Low tot	PND-Low tot	83	17	0	37,405	83	14	0	32,964	45	9	0	35,391	45	9	0	34,694	38	8	0	34,688	38	5	0	27,956
PND-High tot	PND-Low tot	47	17	0	36,532	43	14	0	35,418	26	9	0	43,518	23	9	0	39,456	21	8	0	39,232	20	5	0	32,099

*PND* Prenatal depressive symptoms, *HC* healthy control, *int* internalizing scores, *ext* externalizing scores, *tot* total scores, pa Number of CpGs significant with *p* value adjusted for multiple testing and for sample plate, sentrix position, granulocytes, nucleated red blood cells, cd8t-cells, age at partus, pre-pregnancy BMI, maternal place of birth, parity, gestational age, delivery mode and education, pu Number of CpGs significant with an unadjusted *p* value.

**Table 3:** Specific differentially methylated CpG sites.

Group 1	Group 2	Gene	Location	Probe	Log2 fold change	Mean (SD)	Unadjusted *p* value	Adjusted *p* value	Regulatory feature
Whole study cohort									
PND-High ext	PND-Low ext	Unknown	chr3, 142849987	cg05153152	−0.60	3.06 (0.37) 3.55 (0.57)	4.77E−08	0.038	Unkown
Female offspring									
HC-Low ext	PND-Low ext	Unknown	chr6, 67659438	cg01533057	−1.10	4.14 (0.41) 5.00 (0.42)	4.15E−08	0.034	Unkown
Male offspring									
PND-High int	PND-Low int	Nudix Hydrolase 15 (NUDT15)	chr13, 48610331	cg07027016	−0.72	2.95 (0.30) 3.5 (0.31)	1.36E−08	0.011	Unkown
HC-High ext	HC-Low ext	Tata-Box Binding protein (*TAF10*) and Tripeptidyl Peptidase 1 (*TPP1*)	chr11 6634101	cg00640240	0.45	−4.48 (0.32) −4.86 (0.28)	2.65E−08	0.022	Promoter associated

Location: chr chromosome and genomic position. Groups are based on self-reported prenatal depressive symptoms (PND) or healthy controls (HC) and parental reported child high or low internalizing (int) and externalizing (ext) behavior scores. Group difference in DNA methylation in specific CpGs are presented as log2 fold change (log2 (*M* value group 1/ *M* value group 2)) between the groups, meaning that a negative log2 fold changes means lower DNA methylation in group 1. Mean standard deviation (SD) = mean DNA methylation and SD for group 1, group 2.

In the analysis adjusted for five covariates, there were four genes that were recurrently DM in the different group analysis, presented in Supplementary Table 2, cg19193135 in the CD3G molecule (*CD3G*), cg14275738 in the Sidekick Cell Adhesionb Molecule (*SDK2*), cg25150243 in the Solute Carrier Family 27 Member 2 (*SLC27A2*), and cg06494638 Josephin Domain Containing 2 (*JOSD2*).

### Cord blood DNA methylation in relation to the behavior of the child at 18 months of age

When comparing DNA methylation in relation to internalizing, externalizing, and total CBCL scores without considering maternal mood, no DM CpG sites were identified (Table 2).

### Cord blood DNA methylation differences between the groups based on the behavior of the child at 18 months of age and maternal mood during pregnancy

#### Internalizing problems

There were no DM sites when comparing children exhibiting high with those with low internalizing scores subgroups, in either whole study cohort, or within the PND or HC groups. However, stratification by sex of the child revealed that boys of mothers with PND and those with high CBCL scores for internalizing problems had lower DNA methylation than those with low CBCL scores for internalizing problems in the cg07027016 site of nudix hydrolase 15 (*NUDT15*) (log2 fold change −0.72), shown in Fig. 2, Supplementary Fig. 2, and Table 3. This DM site was nevertheless not evident after exclusion of participants treated with SSRIs. The cg07027016 site DNA methylation was also correlated with continuous CBCL internal scores alone (*β* = 0.080, *p* value 3.20E−228), and also when including EPDS as an interactor (*β* = 0.007, *p* value 2.90E−48), but not when adjusting for any covariates.

#### Externalizing problems

Among the PND participants, we identified one DM site for which those with high CBCL scores for externalizing problems had lower DNA methylation than those with low CBCL scores for externalizing problems. This DM was the cg05153152 (log2 fold change −0.60) and was significant even after exclusion of participants with SSRI. This CpG is located on chromosome 3 at a site without an annotated gene. The cg05153152 site DNA methylation correlated with continuous CBCL external scores (*β* = 0.093, *p* value 1.00E−170) and when EPDS was included as an interactor (*β* = 0.007, *p* value 1.22E−48), but not in the adjusted analysis. Stratification by sex of the child showed that among girls with low CBCL scores for externalizing problems, there was lower DNA methylation in the HC compared to the PND groups at cg01533057 (log2 fold change −1.1), shown in Fig. 2, Supplementary Fig. 2, and Table 3, located on chromosome 6 without an annotated gene. This site was not DM after exclusion of SSRI treated participants. Continuous CBCL external scores were associated with cg01533057 site DNA methylation (*β* = 0.123, *p* value 4.90E−173) and also when including EPDS as an interactor (*β* = 0.010, *p* value 7.75E−46), but not when adjusting for any covariates. Amongst boys born to HC mothers, DNA methylation in the cg00640240 located in the promoter region, associated with the TATA-box binding protein (*TAF10*) and tripeptidyl peptidase 1 (*TPP1*) genes, was higher among those with high CBCL scores for externalizing problems compared to those with low CBCL scores for externalizing problems (log2 fold change 0.45), shown in Fig. 2, Supplementary Fig. 2, and Table 3. This DM site remained significant after exclusion of SSRI treated participants. The cg00640240 site DNA methylation was also correlated with continuous CBCL external scores alone (*β* = −0.011, *p* value 1.52E−47) and when including EPDS as an interactor (*β* = −0.140, *p* value 1.00E−185), but not when adjusted for covariates.

#### Total CBCL score

Groups compared based on PND and their combined CBCL total score did now show any DM sites in any of the analyses (Fig. 2 and Table 2).

### Genes of interest

In addition to genome-wide epigenetic analysis, based on the literature (reviewed in Cao-Lei et al. [27]), CpGs in specific genes were analyzed. There was no DM site in any of these genes among any of the CBCL groups; however, after stratification for child sex, significant DM sites were associated with internalizing scores. Among those girls born to HC mothers, those with high internalizing scores showed higher DNA methylation at cg260557780 in *BDNF* (log2 fold change 0.52), significant also after excluding the SSRI treated participants (log2 fold change 0.51), compared to girls with low internalizing scores. Among boys, after excluding participants using SSRI, we identified a DM site in the cg09080913 CpG in the *MEST* gene where lower DNA methylation was observed in the boys with high internalizing scores born to HC mothers, compared to the boys with low internalizing scores born to PND mothers (log2 fold change −0.34). The same was true for boys with low internalizing scores born to HC mothers, compared to boys with low internalizing scores born to PND mothers (log2 fold change −0.37). Lastly, a difference was also noted in boys with higher internalizing scores compared to those with low internalizing scores (log2 fold change −0.42), among those born to PND mothers.

### Machine learning

Co-variables and DNA methylation in genes of interest did not predict child behavior in any of the machine learning models (logistic regression with lasso regularization, RF, and PLS-DA) implemented in order to try to predict the CBCL internal, external, or total scores in the four study groups or the sub-group analysis. Prediction values ranged between 0.4–0.6, showing that the models cannot predict child behavior at 18 months assessed by use of the CBCL, our outcome of interest, in any of the analysis. Sub-group analyses included CBLC high or low, irrespective of the maternal depression status, or only included children of healthy mothers, or only included mothers with PND, and also testing resilience by including the extreme groups, namely the HC with high CBCL scores together with the PND with low CBCL score groups.

## Discussion

This EWAS study investigated the potential early epigenetic markers of child behavioral problems, assessed using the CBCL on a child aged 18 months, taking into account the maternal PND. Our findings show very few associations between cord blood DNA methylation and child behavior. Generally, our findings point to the absence of differences in methylation between mother-infant pair groups based on exposure to maternal depression and later presence of behavioral problem scores. While studies on maternal PND and child development show increased risks for children exposed to PND [6, 7], our findings concerning DNA methylation taking into account maternal PND are in agreement with most previous studies [15, 28, 49]. This study is, however, the first investigation combining rigorous assessment of depressive symptoms during pregnancy and postpartum, an EWAS analysis of umbilical cord blood, and behavioral follow-up at 18 months of age. The results are strengthened by the exclusion of mothers with postpartum depression, which allowed us to focus solely on possible intrauterine impact and also to avoid a state-effect when mothers report on their children’s behavior.

Prenatal depressive symptoms have been associated with DM of target genes [16, 17, 27]; moreover, we have previously shown an association between PND in mothers and higher CBCL scores in children, suggesting the possibility of fetal programming [7]. The current study, however, shows limited indications of fetal programming through epigenetic changes in the cord blood, since only a few DM CpG sites were identified in subgroups displaying different levels of psycho-emotional problems as toddlers, especially by the infant’s sex. It must be noted that the present study might differ from other studies as it is based on a relatively healthy group of mother-child pairs in Sweden [33].

To consider the impact of technical and clinical variables, the analyses were performed while adjusting for potential covariates. In the results from the analysis adjusted from the five technical covariates, there were genes that were DM in several group comparisons. Interestingly, some of them, such as *CD3G* and *SLC27A2*, have been shown to be associated with maternal obesity during pregnancy [50, 51]. These DMs are no longer evident after adjusting for maternal BMI and other factors known to be associated with DNA methylation in cord blood. Therefore, the main results presented in this study are focused on results obtained after adjusting for 12 covariates associated with the DNA methylation [15, 26, 43–45].

In the analysis adjusted for the 12 covariates, including both boys and girls, only one DM CpG site, cg05153152, on chromosome 3, with no annotated gene, was identified when comparing children with high externalizing problem scores to those with low externalizing scores, all born to mothers suffering PND. The unannotated site makes it challenging to speculate on the importance of DM cg05153152. This site may, however, have a potential role for resilience in child development in relation to exposure to prenatal depressive symptoms and should be validated in future studies. One unannotated CpG site, cg01533057, was also identified on chromosome 6 with no annotated gene in the stratified analysis in girls when comparing girls with low externalizing scores, born to HC mothers, to those born to mothers with PND. Without annotation, it is difficult to speculate on the importance of this DM CpG site.

Stratified analyses based on the sex of the child revealed some DM sites in boys with regard to maternal depressive symptoms and internalizing and externalizing behavioral problems. Among boys born to women with PND, those with high internalizing scores had lower methylation at cg07027016 in *NUDT15* (also known as MTH2) compared to those with low internalizing scores. In a similar matter as cg05153152, the DM cg07027016 in *NUDT15* may be of importance for resilience in child development in relation to exposure to prenatal depressive symptoms. *NUDT15* has not previously been discussed in the context of PND or child development, but it has been shown to be involved in DNA synthesis and cell cycle progression [52] and to be expressed in the brain (https://www.proteinatlas.org/ENSG00000136159-NUDT15/tissue). It could, therefore, be important for cognitive or emotional functioning [53] and suggests a possible role in fetal brain development in boys during pregnancy. This is especially interesting, as previous literature indicates sex specific effects of PND. While most studies have indicated a greater impact on the fetal brain in females (reviewed in [54]), later cognitive functioning and IQ are suggested to be more affected in boys during adolescence [23]. Also, in boys, *MEST* (or *PEG1*) had one DM CpG site after exclusion of SSRI-treated participants. The PND with the low internalizing score group showed a higher DNA methylation compared to the HC with high internalizing, HC with low internalizing, and PND with high internalizing score groups. Lower DNA methylation in *MEST* is suggested to contribute to obesity predisposition [55]. Notably, altered DNA methylation of this gene in cord blood has previously been associated with maternal stress during pregnancy [56]; nevertheless, a previous study reported no association when investigating maternal depressive symptoms [27, 57].

Of special interest is the higher level of DNA methylation observed among HC mothers at cg00640240, associated with the promoter region of *TPP1* and *TAF10*, boys with high externalizing scores compared to those with low externalizing scores. These boys have not been exposed to maternal depressive symptoms but still have higher externalizing scores. Interestingly, mutations in *TPP1* are one of the causes of late infantile neuronal ceroid lipofuscinosis, a fatal neurodegenerative disease [58] associated with altered externalizing behavior alongside neurodegeneration [59]. One may speculate that differential expression due to altered DNA methylation in the promoter region of *TPP1* may be important for the mechanisms contributing to problems in externalizing behavior, in the absence of extra risk factors, such as maternal depression. *TAF10* is not associated with any disorders but is vital for cellular transcription initiation [60] and is involved in neural progenitor differentiation [61].

The investigation of genes identified in the literature, where the *p* value adjustment for multiple comparisons was based on the number of CpGs in the genes of interest, instead of all included in the EPIC array, showed significant DM sites only after stratification by the sex of the child. The CpG site in *BDNF* was DM in girls but only among those of healthy mothers, where a higher methylation was observed in girls with high internalizing scores. Interestingly, peripheral BDNF levels have previously been shown to correlate positively with CBCL scores [62]. Furthermore, BDNF is an important factor for survival of neurons by regulating growth, maturation, and maintenance [27], as well as in depressive symptoms in adult women [63] and could thus have a possible involvement mechanism behind internalizing behavioral problems. Machine learning models used to investigate possible markers of resilience in genes previously identified in the literature did not show any predicative function. Of special interest, PND low CBCL vs. PND high CBCL, focusing on only children exposed to PND, did not show any predictive ability either, prediction values ranging between 0.4–0.6 in the machine learning analyses.

Three DM CpG sites in the *TPP1, TAF10*, and *BDNF* genes were associated with externalizing and internalizing scores in children not exposed to maternal depressive symptoms. These findings are of great interest since they may be validated in other study cohorts and possibly used as a biomarker for behavioral disorders later in life, irrespective of the prenatal exposure to maternal mood symptoms. The prospective role of resilience identified in cg05153152 and also in cg07027016 in the *NUDT15* gene should also be validated in future studies as markers for resilience.

We have previously shown differentially methylated genes in cord blood associated with maternal SSRI use during pregnancy [29]. It was not possible, however, to assess if these methylation differences were due to the SSRI treatment itself or to more severe depression. Nonetheless, in this study, we excluded SSRI users in the sensitivity analyses, which affected the results in some of the identified genes. This strengthens our previous findings [29] that more severe depression, in need of SSRI treatment during pregnancy, may be associated with alterations in the DNA methylation and is of importance for the regulation of biological systems in the child. Unfortunately, due to the limited number of women under SSRI treatment during pregnancy in this specific study (*n* = 7 in total but spread through groups of interest), no separate analyses in relation to child behavior could be performed in this group.

Strengths of the present study are the use of a population-based sample, the relatively large sample size for the EWAS analyses, the prospective assessment of PND, exclusion of mothers with postpartum depression in order to focus solely on the intrauterine impact and avoid a state-effect when reporting on children’s behavior, EWAS analysis of the cord blood, and longitudinal follow-up of child behavior. Moreover, weaknesses of previous studies were addressed in the current study by adjustment for mode of delivery [43], gestational age [44], and cell composition known to affect DNA methylation [24, 25], as well as the exclusion of participants with pregnancy complications, also known to affect DNA methylation [64, 65]. Further, we have implemented controls for multiple testing, stratified our analyses by sex, excluded users of SSRI in the sensitivity analyses, and used complex machine learning models. The results should also be considered in relation to the following limitations. The BASIC study has a relatively low participation rate at 22% [33]; thus, the results cannot be readily generalizable to the background population. Depressive symptoms were assessed through self-reports on a screening instrument and not by a clinical diagnosis, rendering even milder cases of depression PND cases in this study. CBCL was parent-reported; no clinical assessment of children’s behavior was implemented. Also, our study population is healthy in relation to both levels of maternal depression and child outcomes. None of the children in this study would have been clinically diagnosed with an internalizing or externalizing disorder if clinical CBCL cut-offs were used for the diagnosis. In addition, the magnitude of differences in methylation levels was small between the groups. The use of surrogate tissue is a limitation, and one major challenge in psychological epigenetics is the cell/tissue-specificity of epigenetic patterns. However, cell-specific analysis would limit research to certain brain areas of suicide victims or cadavers and thereby limit the possibility of investigating early biomarkers affected by maternal depressive symptoms. The use of cell composition correction presented by Bakulski et al. has not been validated for samples with different exposures. The validity of the Bakulski package for different types of prenatal exposures can only be tested if samples were collected and cells separated at sampling and DNA methylation analysis performed on the whole blood and separated cell types and compared regarding the prenatal exposure. That is unfortunately not possible within this study cohort. The identified DM sites in this study have not previously been shown to be listed as cross-reactive probes [66]; however, not excluding these during the analysis can cause false signals, possibly resulting in invalid conclusions, but our DM CpGs were not included in the list of genes previously identified as cross-reactive probes [66], which should account for this limitation. We did, however, exclude known SNPs, which strengthened our data. This study excluded sex chromosomes in order to be able to perform differential DNA methylation analysis between the study groups, usually performed in this context [42]. This is a limitation, since many known genes affecting mental health are located on the X chromosome [67]. Future studies should be encouraged to increase the sample size and thus power, in order to also be able to investigate the DNA methylation in sex chromosomes, among same sex children. Upcoming studies may also further investigate the impact of epigenetic markers in cord blood on the development of behavioral problems in children by use of complementary epigenetic analyses focusing on histone modifications and considering transcriptional factor binding sites.

In conclusion, this study shows CpG-specific DNA methylation differences in cord blood at birth as early signs of later behavior problems, in both healthy pregnancies and those complicated by prenatal depression. Notably, there is limited knowledge of how many DM sites are needed to influence the biological and behavioral outcomes. The present findings are based on a relatively healthy study cohort, thus limiting the possibility of detecting strong effects associated with severe prenatal depression and pathological behavioral problems. Moreover, it is likely that fetal programming develops through the interplay between multiple epigenetic mechanisms that were not assessed here, such as histone modifications or microRNAs affecting gene expression, alternatively, through DNA methylation on CpG sites not investigated in the EPIC array. Altogether, this is the first investigation of cord blood DNA methylation signs of fetal programming of prenatal depressive symptoms on behavior at 18 months of age in a population of rather healthy children. Thus, this calls for further studies on samples covering the entire behavioral spectrum.

## Supplementary information

Supplementary figure legends

Supplementary table 1

Supplementary table 2

Supplementary figure 1

Supplementary figure 2

## Data Availability

Currently, the General Data Protection Regulation (GDPR), and the judgment in the Court of Justice of the European Union (CJEU) Decision 2016/1250, hinder data deposition of human genetic data. The data and code used in this study are available from the authors upon reasonable request and with permission from the BASIC study cohort.
