# Supplementary material for: Maternal prenatal depressive symptoms and toddler behavior: an umbilical cord blood epigenome-wide association study
Source: Transl Psychiatry. 2022 May 5;12:186. doi: 10.1038/s41398-022-01954-6 (PMC9072531; doi:10.1038/s41398-022-01954-6)
Supplement: Supplementary file 1 — Supplementary figure legends [file 41398_2022_1954_MOESM1_ESM.docx]

**Supplementary figure legends**

**Supplementary Figure 1.** Quantile-quantile plot (QQ-plot) displaying the observed and expected Benjamini-Hochberg adjusted –log_10_ p-values for group comparisons, where significant differentially methylated sites were identified.

Groups were based on self-reported prenatal depressive symptoms (PND) or healthy controls (HC) and parental reported child internalizing and externalizing behavior scores.

1. PND with high externalizing scores compared to PND with low externalizing scores
2. PND with high externalizing scores compared to PND with low externalizing scores, excluding children exposed to selective serotonin reuptake inhibitors.
3. HC with low externalizing scores compared to PND with low externalizing scores in female children.
4. PND with high internalizing scores compared to PND with low internalizing scores in male children.
5. PND with high internalizing scores compared to PND with low internalizing scores in male children.
6. PND with high internalizing scores compared to PND with low internalizing scores in male children, excluding children exposed to selective serotonin reuptake inhibitors.

**Supplementary Figure 2.** Manhattan plots displaying differentially DNA methylated genes in the cord blood. Y-axes display the Benjamini-Hochberg adjusted –log_10_ p-values for specific group comparisons and x-axes show the chromosomal location.

Groups were based on self-reported prenatal depressive symptoms (PND) or healthy controls (HC) and parental reported child internalizing and externalizing behavior scores.

1. PND with high externalizing scores compared to PND with low externalizing scores
2. PND with high externalizing scores compared to PND with low externalizing scores, excluding children exposed to selective serotonin reuptake inhibitors.
3. HC with low externalizing scores compared to PND with low externalizing scores in female children.
4. PND with high internalizing scores compared to PND with low internalizing scores in male children.
5. HC with high externalizing scores compared to HC with low externalizing scores in male children.
6. HC with high externalizing scores compared to HC with low externalizing scores in male children, excluding children exposed to selective serotonin reuptake inhibitors.
