# Supplementary material for: Maternal prenatal depressive symptoms and toddler behavior: an umbilical cord blood epigenome-wide association study
Source: Transl Psychiatry. 2022 May 5;12:186. doi: 10.1038/s41398-022-01954-6 (PMC9072531; doi:10.1038/s41398-022-01954-6)
Supplement: Supplementary file 2 — Supplementary table 1 [file 41398_2022_1954_MOESM2_ESM.pdf]

Supplementary table 1. Group comparisons adjusted for 0, 5, or 12 covariates.

		Whole																							
Number of covariates		0								5								12							
		All				SSRI-treatment excluded				All				SSRI-treatment excluded				All				SSRI-treatment excluded			
Group 1	Group 2	n1	n2	pa	pu	n1	n2	pa	pu	n1	n2	pa	pu	n1	n2	pa	pu	n1	n2	pa	pu	n1	n2	pa	pu
High int	Low int	140	116	0	42123	136	113	0	45729	140	116	0	50274	136	113	0	52218	132	115	0	47111	128	112	0	47505
HC-High int	HC-Low int	90	92	0	32910	90	92	0	33273	90	92	0	39809	90	92	0	40360	89	92	0	40165	89	92	0	40506
HC-High int	PND-High int	90	50	0	46655	90	46	0	46928	90	50	0	38787	90	46	0	38197	89	43	0	44070	89	39	0	41104
HC-High int	PND-Low int	90	24	0	37535	90	21	0	40883	90	24	0	60377	90	21	0	58285	89	23	0	71787	89	20	0	67190
HC-Low int	PND-High int	92	50	0	51947	92	46	0	52391	92	50	0	40267	92	46	0	41655	92	43	0	37819	92	39	0	37534
HC-Low int	PND-Low int	92	24	0	33035	92	21	0	33186	92	24	0	44709	92	21	0	43357	92	23	0	51457	92	20	0	49173
PND-High int	PND-Low int	50	24	0	44788	46	21	1	51819	50	24	0	52795	46	21	1	55622	43	23	0	50610	39	20	0	52091
Hight ext	Low ext	138	101	0	77149	132	99	0	69716	138	101	0	34530	132	99	0	34910	134	97	0	37800	128	95	0	38268
HC-High ext	HC-Low ext	86	80	0	46384	86	80	0	46790	86	80	0	42372	86	80	0	42587	85	80	0	44378	85	80	0	44866
HC-High ext	PND-High ext	86	52	0	41684	86	46	1	37423	86	52	0	52551	86	46	0	46595	85	49	0	69903	85	43	0	59957
HC-High ext	PND-Low ext	86	21	1	64092	86	19	1	58484	86	21	0	41548	86	19	0	40343	85	17	0	37679	85	15	0	35903
HC-Low ext	PND-High ext	80	52	0	67188	80	46	0	57289	80	52	0	40897	80	46	0	37643	80	49	0	50085	80	43	0	44444
HC-Low ext	PND-Low ext	80	21	0	53244	80	19	4	48829	80	21	0	53560	80	19	4	51113	80	17	0	42682	80	15	0	40010
PND-High ext	PND-Low ext	52	21	16	83375	46	19	4	69748	52	21	0	51843	46	19	0	48970	49	17	1	48940	43	15	1	45621
High tot	Low tot	142	102	0	66742	138	99	0	60338	142	102	0	34174	138	99	0	34748	136	100	0	37124	132	97	0	37850
HC-High tot	HC-Low tot	90	83	0	42100	90	83	0	42129	90	83	0	40140	90	83	0	40535	89	83	0	42788	89	83	0	43490
HC-High tot	PND-High tot	90	52	0	36447	90	48	0	35696	90	52	0	47361	90	48	0	44179	89	47	0	62034	89	43	0	55409
HC-High tot	PND-Low tot	90	19	0	64404	90	16	0	51838	90	19	0	38674	90	16	0	35455	89	17	0	42263	89	14	0	35599
HC-Low tot	PND-High tot	83	52	0	52282	83	48	0	49847	83	52	0	37424	83	48	0	36255	83	47	0	42190	83	43	0	39766
HC-Low tot	PND-Low tot	83	19	0	46810	83	16	1	39852	83	19	0	41100	83	16	1	38679	83	17	0	37405	83	14	0	32964
PND-High tot	PND-Low tot	52	19	3	70338	48	16	0	56456	52	19	0	37373	48	16	0	37198	47	17	0	36532	43	14	0	35418

		Female																							
Number of covariates		0								5								12							
		All				SSRI-treatment excluded				All				SSRI-treatment excluded				All				SSRI-treatment excluded			
Group 1	Group 2	n1	n2	pa	pu	n1	n2	pa	pu	n1	n2	pa	pu	n1	n2	pa	pu	n1	n2	pa	pu	n1	n2	pa	pu
High int	Low int	73	59	0	30748	70	58	0	34535	73	59	0	37869	70	58	0	39495	70	59	0	34518	67	58	0	34590
HC-High int	HC-Low int	43	49	0	35434	43	49	0	35502	43	49	0	35152	43	49	0	34754	43	49	0	33980	43	49	0	33361
HC-High int	PND-High int	43	30	0	39977	43	27	0	42111	43	30	0	33949	43	27	0	34796	43	27	0	35021	43	24	0	33855
HC-High int	PND-Low int	43	10	0	34651	43	9	0	35601	43	10	0	38216	43	9	0	38177	43	10	0	42357	43	9	0	39869
HC-Low int	PND-High int	49	30	0	32744	49	27	0	34635	49	30	0	35868	49	27	0	38031	49	27	0	33217	49	24	0	33340
HC-Low int	PND-Low int	49	10	0	31867	49	9	0	29003	49	10	0	34463	49	9	0	34902	49	10	0	38283	49	9	0	37605
PND-High int	PND-Low int	30	10	0	27715	27	9	0	32151	30	10	0	36010	27	9	0	39902	27	10	0	36241	24	9	0	37936
Hight ext	Low ext	65	60	1	63075	61	60	0	54341	65	60	0	40182	61	60	0	38144	64	58	0	43770	60	58	0	41719
HC-High ext	HC-Low ext	37	49	0	34957	37	49	0	34964	37	49	0	40566	37	49	0	40746	37	49	0	40449	37	49	0	40883
HC-High ext	PND-High ext	37	28	0	38179	37	24	0	33234	37	28	0	46281	37	24	0	43510	37	27	0	53075	37	23	0	44726
HC-High ext	PND-Low ext	37	11	1	65650	37	11	0	65691	37	11	2	52204	37	11	0	49526	37	9	0	39569	37	9	0	38231
HC-Low ext	PND-High ext	49	28	0	57625	49	24	0	45203	49	28	0	40621	49	24	0	37538	49	27	0	48901	49	23	0	42005
HC-Low ext	PND-Low ext	49	11	0	58460	49	11	0	58297	49	11	1	62081	49	11	1	58296	49	9	1	40581	49	9	0	37974
PND-High ext	PND-Low ext	28	11	2	88485	24	11	1	76946	28	11	0	74572	24	11	0	65903	27	9	0	60152	23	9	0	49011
High tot	Low tot	69	55	0	43643	66	55	0	42469	69	55	0	34350	66	55	0	33814	67	54	0	37030	64	54	0	35779
HC-High tot	HC-Low tot	41	45	0	34789	41	45	0	34386	41	45	0	35376	41	45	0	35643	41	45	0	33911	41	45	0	34068
HC-High tot	PND-High tot	41	28	0	36871	41	25	0	34966	41	28	0	39150	41	25	0	37643	41	26	0	42880	41	23	0	38093
HC-High tot	PND-Low tot	41	10	0	62742	41	10	0	62124	41	10	0	44616	41	10	0	43385	41	9	0	35517	41	9	0	35286
HC-Low tot	PND-High tot	45	28	0	35024	45	25	0	33658	45	28	0	34530	45	25	0	33603	45	26	0	41780	45	23	0	37415
HC-Low tot	PND-Low tot	45	10	5	53193	45	10	4	52758	45	10	1	50755	45	10	1	48695	45	9	0	35391	45	9	0	34694
PND-High tot	PND-Low tot	28	10	0	66370	25	10	0	62953	28	10	0	54482	25	10	0	49993	26	9	0	43518	23	9	0	39456

		Male																							
Number of covariates		0								5								12							
		All				SSRI-treatment excluded				All				SSRI-treatment excluded				All				SSRI-treatment excluded			
Group 1	Group 2	n1	n2	pa	pu	n1	n2	pa	pu	n1	n2	pa	pu	n1	n2	pa	pu	n1	n2	pa	pu	n1	n2	pa	pu
High int	Low int	67	57	0	58174	66	55	0	52160	67	57	0	43280	66	55	0	42770	62	56	0	47403	61	54	0	46870
HC-High int	HC-Low int	47	43	0	36799	47	43	0	37228	47	43	0	36731	47	43	0	37042	46	43	0	39394	46	43	0	40542
HC-High int	PND-High int	47	20	0	39381	47	19	0	35783	47	20	0	43892	47	19	0	42798	46	16	0	44308	46	15	0	42135
HC-High int	PND-Low int	47	14	0	39755	47	12	0	36279	47	14	0	44343	47	12	0	43879	46	13	0	47373	46	11	0	45661
HC-Low int	PND-High int	43	20	0	58075	43	19	0	49571	43	20	1	44911	43	19	0	43338	43	16	0	44808	43	15	0	42288
HC-Low int	PND-Low int	43	14	0	31559	43	12	0	29625	43	14	0	38038	43	12	0	37506	43	13	0	38183	43	11	0	36319
PND-High int	PND-Low int	20	14	0	57413	19	12	0	47703	20	14	0	47281	19	12	0	46073	16	13	1	49576	15	11	0	45477
Hight ext	Low ext	73	41	0	45144	71	39	0	42639	73	41	0	34860	71	39	0	35282	70	39	0	39338	68	37	0	40561
HC-High ext	HC-Low ext	49	31	0	36980	49	31	0	37403	49	31	1	38963	49	31	0	38443	48	31	1	46230	48	31	1	46992
HC-High ext	PND-High ext	49	24	0	33093	49	22	0	33480	49	24	0	44759	49	22	0	42367	48	22	0	50427	48	20	0	46387
HC-High ext	PND-Low ext	49	10	0	48109	49	8	0	38420	49	10	0	40555	49	8	0	38574	48	8	0	38968	48	6	0	35516
HC-Low ext	PND-High ext	31	24	0	37906	31	22	0	37193	31	24	0	35491	31	22	0	35258	31	22	0	36059	31	20	0	35834
HC-Low ext	PND-Low ext	31	10	0	44463	31	8	0	36579	31	10	1	39221	31	8	0	37026	31	8	0	38662	31	6	0	35611
PND-High ext	PND-Low ext	24	10	0	53136	22	8	0	44321	24	10	0	37810	22	8	0	35613	22	8	0	40102	20	6	0	37058
High tot	Low tot	73	47	0	86820	72	44	0	73903	73	47	0	36637	72	44	0	35190	69	46	0	44327	68	43	0	41705
HC-High tot	HC-Low tot	49	38	0	58421	49	38	0	58703	49	38	0	36425	49	38	0	36405	48	38	0	43278	48	38	0	43599
HC-High tot	PND-High tot	49	24	0	33631	49	23	0	32690	49	24	0	43960	49	23	0	43544	48	21	0	46215	48	20	0	45373
HC-High tot	PND-Low tot	49	9	7	61284	49	6	12	35683	49	9	4	37049	49	6	4	30242	48	8	0	41582	48	5	0	29787
HC-Low tot	PND-High tot	38	24	0	59926	38	23	1	51858	38	24	0	39562	38	23	0	39127	38	21	0	40619	38	20	0	40074
HC-Low tot	PND-Low tot	38	9	2	41878	38	6	4	27842	38	9	0	34033	38	6	2	29496	38	8	0	34688	38	5	0	27956
PND-High tot	PND-Low tot	24	9	2	69093	23	6	6	38588	24	9	1	35739	23	6	3	31079	21	8	0	39232	20	5	0	32099

Abbreviations: prenatal depressive symptoms (PND), Healthy control (HC), internalizing scores (int), externalizing scores (ext), total scores (tot), pa = number of CpGs significant with p-value adjusted for multiple testing 0 covariates, for 5 technical covariates (sample plate, sentrix position, granulocytes, nucleated red blood cells, cd8t-cells) or 12 covariates (technical plus age at partus, pre-pregnancy bmi, maternal place of birth, pari gestational age, delivery mode and education) and finally pu = number of CpGs p-value unadjusted.
