# Supplementary material for: Maternal prenatal depressive symptoms and toddler behavior: an umbilical cord blood epigenome-wide association study
Source: Transl Psychiatry. 2022 May 5;12:186. doi: 10.1038/s41398-022-01954-6 (PMC9072531; doi:10.1038/s41398-022-01954-6)
Supplement: Supplementary file 3 — Supplementary table 2 [file 41398_2022_1954_MOESM3_ESM.pdf]

Supplementary table 2. CpG sites identified when only adjusting for technical covariates.

Group 1	Group 2	Gene	Location	Probe	Log2 foldchange	Unadjusted p-value	Adjusted p-value	Regulatory feature
*Whole study cohort**								
PND-High int	PND-Low int	Kelch Like Family Member 20	chr.1 173683625	cg17859089	-0.64	1.811E-08	0.015	Promoter associated
HC-Low ext	PND-Low ext	Hydroxysteroid Dehydrogenase Like 1	chr.16 84178939	cg03097336	0.44	2.222E-07	0.049	Promoter associated
		Otoferlin	chr.2 26718652	cg07892276	-0.36	2.404E-07	0.049	Unknown
		Mediator Complex Subunit 11	chr.17 4634072	cg11361925	0.46	1.7167E-07	0.049	Unknown
		CD3G Molecule	chr. 11 118219874	cg19193135	0.57	1.745E-07	0.049	Unknown
HC-Low tot	PND-Low tot	CD3G Molecule	chr. 11 118219874	cg19193135	-0.67	3.030E-8	0.025	Unknown
Female offspring								
HC-High ext	PND-Low ext	Zinc Finger And BTB Domain Containing 8B	chr.1 32953089	cg07019697	0.46	9.95E-08	0.042	Unknown
		CD3G Molecule	chr. 11 118219874	cg19193135	0.90	1.03E-07	0.042	Unknown
HC-Low ext	PND-Low ext	CD3G Molecule	chr. 11 118219874	cg19193135	0.97	2.18E-09	0.001	Unknown
HC-Low tot	PND-Low tot	CD3G Molecule	chr. 11 118219874	cg19193135	1.00	2.66E-09	0.002	Unknown
Male offspring								
HC-Low int	PND-High int	Small Glutamine Rich Tetratricopeptide Repeat Co-Chaperone Beta; Neurolysin	chr. 5 65046588	cg09873510	0.61	3.82E-08	0.031	Unknown
HC-High ext	HC-Low ext	Tata-Box Binding protein; Tripeptidyl Peptidase 1	chr11 6634101	cg00640240	0.42	5.75E-08	0.047	Promoter associated
HC-Low ext	PND-Low ext	Transmembrane Protein 86B; Protein Phosphatase 6 Regulatory Subunit 1	chr.19 55741371	cg13198182	1.00	4.68E-08	0.038	Unknown
HC-High tot	PND-Low tot	Bardet-Biedl Syndrome 4	chr. 15 73015041	cg12429188	-1.47	4.56E-08	0.019	Unclassified
		Sidekick Cell Adhesion Molecule 2	chr.17 71640340	cg14275738	0.97	2.40-08	0.019	Unknown
			chr.12 15409058	cg06157779	-1.01	9.04E-08	0.025	Unknown
		Component Of Oligomeric Golgi Complex 5	chr. 7 107204581	cg06080224	1.00	2.19E-07	0.045	Promoter associated
HC-Low tot	PND-Low tot*	Solute Carrier Family 27 Member 2	chr.15 50474159	cg25150243	1.62	1.84E-08	0.015	Promoter associated
		Josephin Domain Containing 2	chr.19 51010382	cg06194638	-1.55	1.06E-07	0.043	Unknown
PND-High tot	PND-Low tot	Sidekick Cell Adhesion Molecule 2	chr.17 71640340	cg14275738	1.13	6.07E-09	0.005	Unknown
PND-High tot	PND-Low tot*	Solute Carrier Family 27 Member 2	chr.15 50474159	cg25150243	1.69	2.26E-08	0.018	Promoter associated
		Small Nuclear Ribonucleoprotein Polypeptide E	chr.1 203831354	cg23316017	-1.74	1.07E-07	0.044	Unknown
		Josephin Domain Containing 2	chr.19 51010382	cg06194638	-1.60	1.64E-07	0.045	Unknown

Location: chromosome and genomic position. Groups are based on self-reported prenatal depressive symptoms (PND) or healthy controls (HC) and parental reported child high or low internalizing (int) and externalizing (ext) behavior scores. Group difference in DNA methylation in specific CpGs are presented as log2 fold change (log2 (M-value group1/M-value group 2)) between the groups, meaning that a negative log2 fold changes means lower DNA methylation in group 1. Abbreviations: chromosome (chr), standard deviation (SD). *Selective serotonin reuptake inhibitors treatment excluded.
