# Supplementary figures and images for: Maternal prenatal depressive symptoms and toddler behavior: an umbilical cord blood epigenome-wide association study
Source: Transl Psychiatry. 2022 May 5;12:186. doi: 10.1038/s41398-022-01954-6 (PMC9072531; doi:10.1038/s41398-022-01954-6)

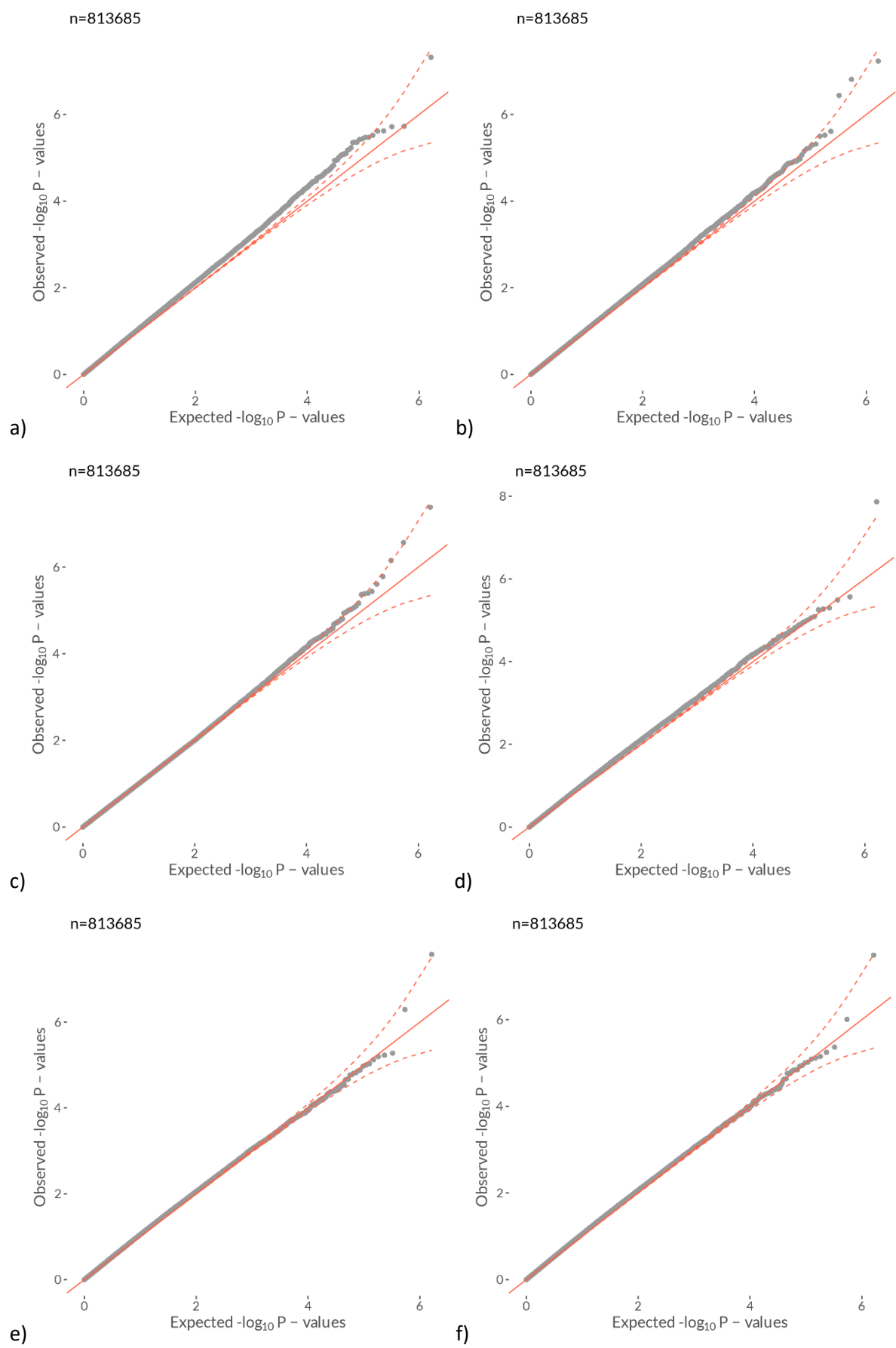

Supplement: Supplementary file 4 — Supplementary figure 1 [file 41398_2022_1954_MOESM4_ESM.pdf]

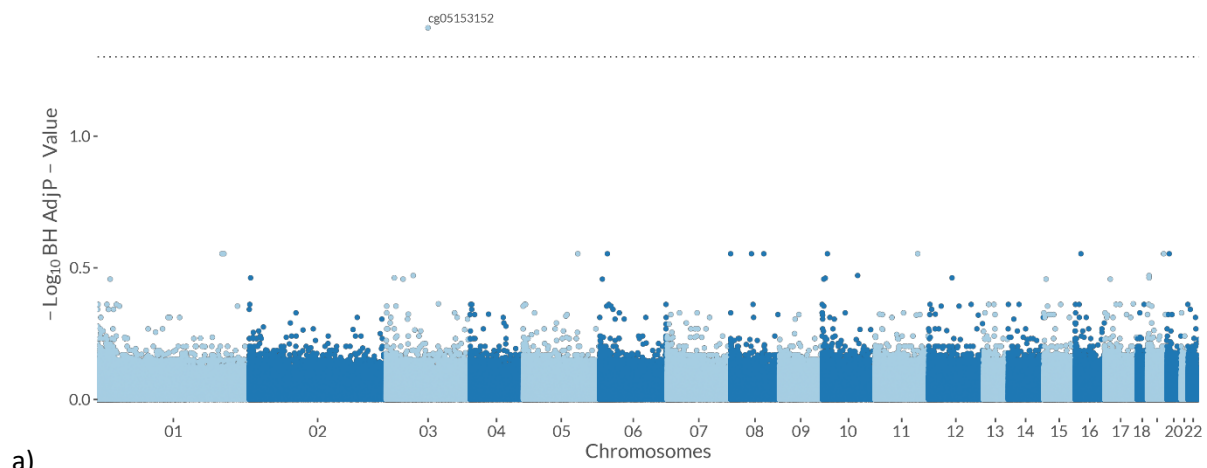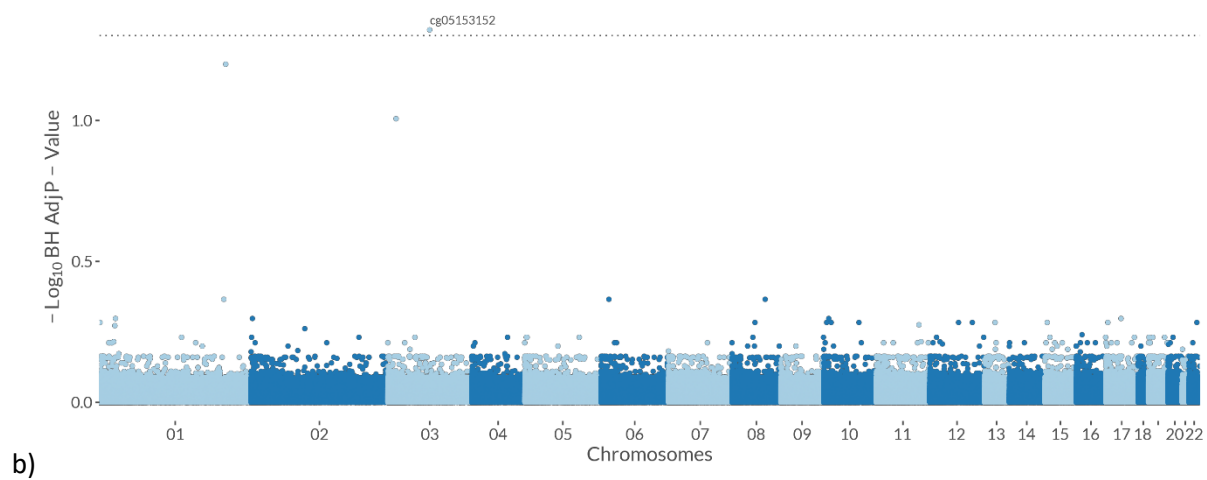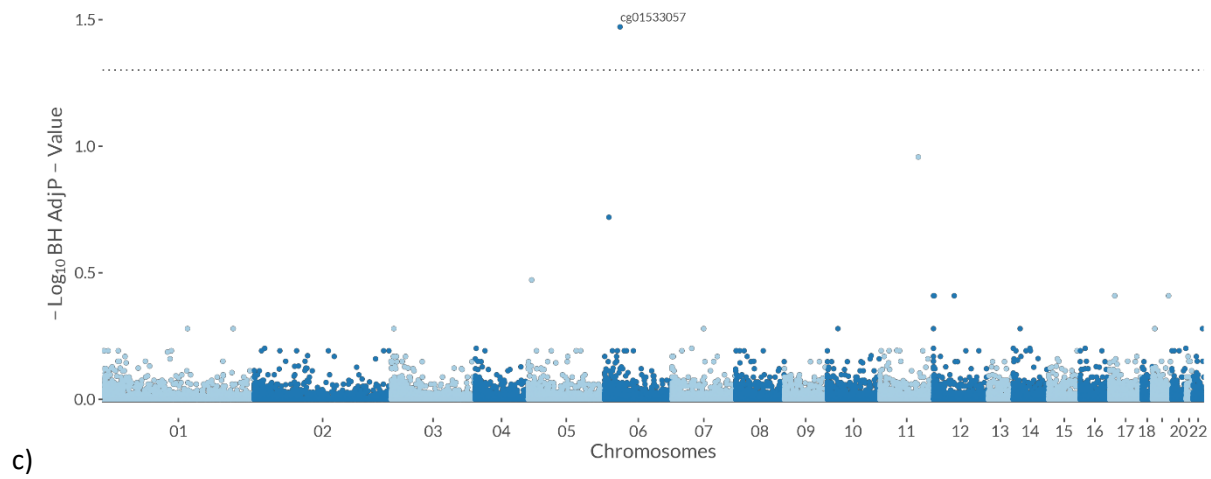

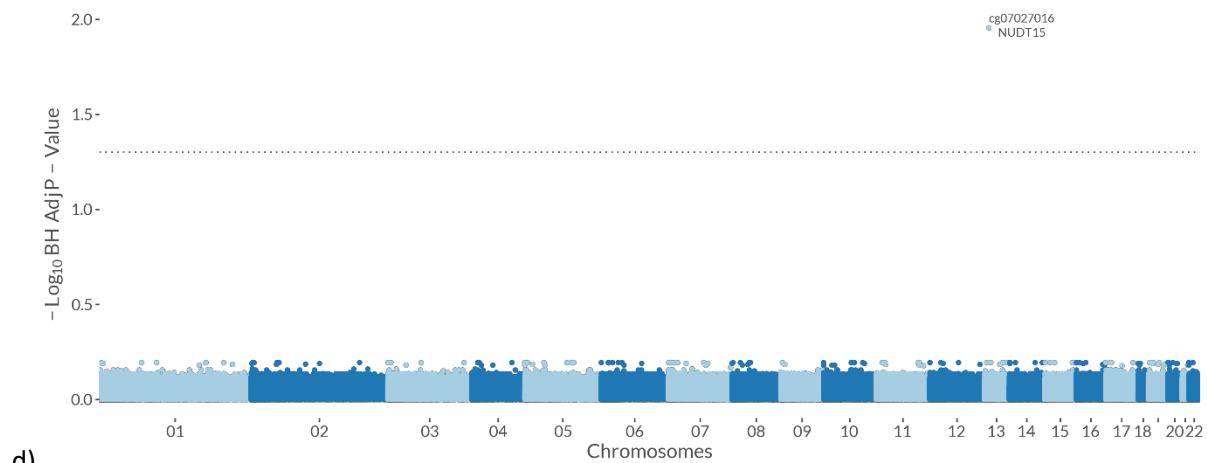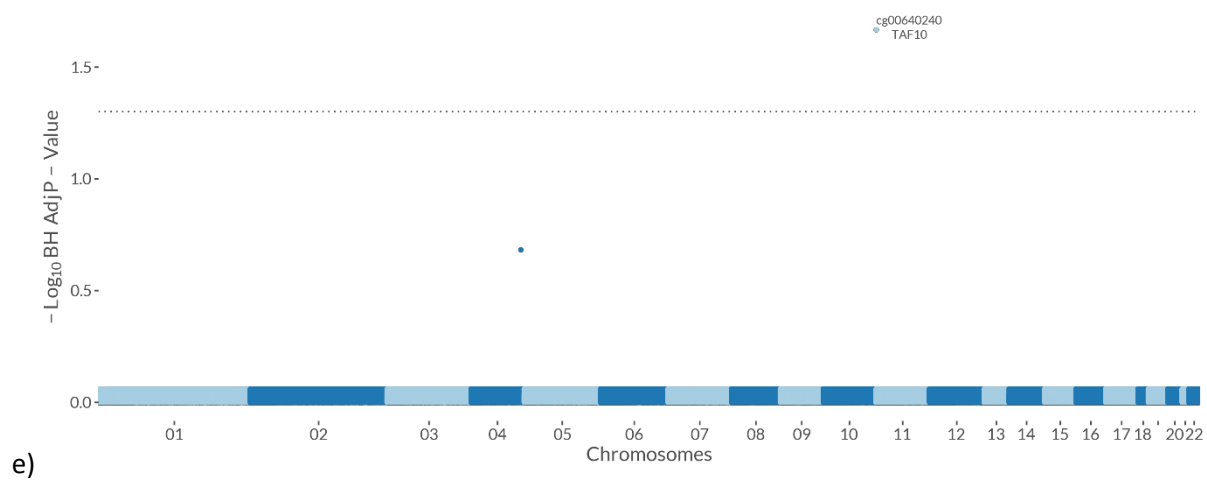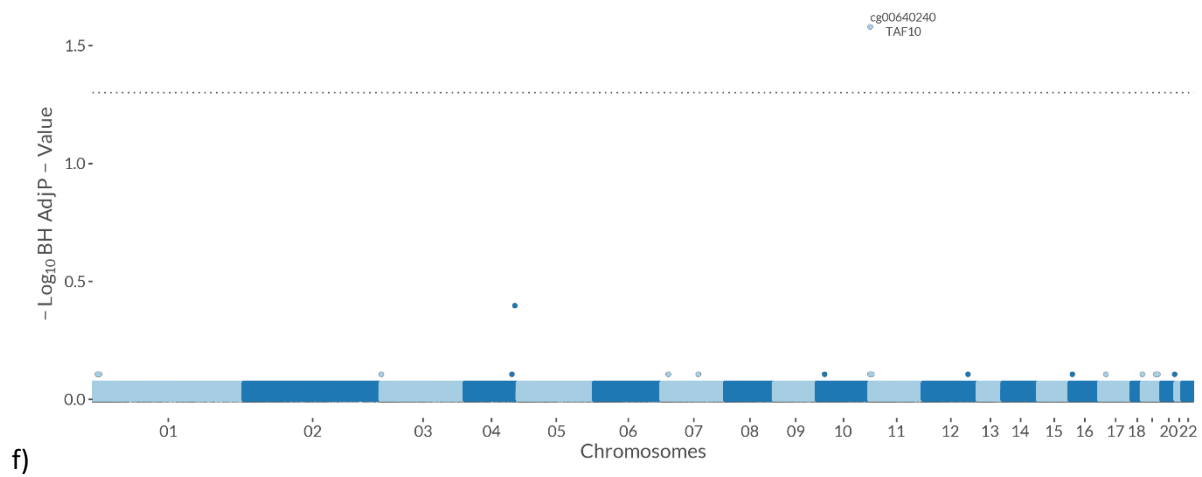

Supplement: Supplementary file 5 — Supplementary figure 2 [file 41398_2022_1954_MOESM5_ESM.pdf]
